# Clinical study on the treatment of adolescent idiopathic scoliosis by balanced jar therapy with flexible corrective appropriate technology

**DOI:** 10.3389/fmed.2026.1716190

**Published:** 2026-03-16

**Authors:** Fen He, Yongqi Xie, Haifeng Zhu, Yuheng Feng, Changqing Ye, Honghu Li, Zhijun Wang, Lilei He

**Affiliations:** 1 The Eighth Clinical Medical College of Guangzhou University of Chinese Medicine, Foshan, Guangdong, China; 2 Foshan Hospital of Traditional Chinese Medicine, Foshan, Guangdong, China

**Keywords:** adolescent idiopathic scoliosis, balanced jar therapy, clinical outcomes, flexible corrective appropriate technology, gait and plantar pressure, quality of life

## Abstract

**Aim:**

The aim of this study was to investigate the efficacy of balanced jar therapy combined with the Flexible Corrective Appropriate Technology (FCAT) in adolescent idiopathic scoliosis (AIS).

**Methods:**

This randomized, single-blind study enrolled adolescents with AIS from October 2022 to September 2024. Participants were divided into control and observation groups. The control group received flexible bracing for at least 6 months, while the observation group received an additional 2-week course of balanced cupping therapy-including flash, kneading, pushing, retention, and removal cupping techniques. Primary outcomes, which include the reduction in Cobb angle and apical transverse rotation (ATR), were assessed at 3- and 6-months post-intervention. Secondary outcomes, including gait parameters, plantar pressure measures, spinal mobility, adverse events, and quality of life, were also evaluated at the same timepoints.

**Results:**

The observation group demonstrated significantly greater reduction in Cobb angle (24.16 ± 3.25 vs. 26.98 ± 3.87, *P* = 0.007) and ATR (10.24 ± 1.66 vs. 11.79 ± 1.83, *P* = 0.003) at 3 months. The plantar pressure indices, particularly the center of pressure excursion index (CPEI), showed significantly greater improvement in the observation group (8.87 ± 2.17 vs. 10.54 ± 2.21, *P* = 0.020). Changes in apical vertebral rotation (AVR) (*P* = 0.001) and the distance of the apical vertebral translation (AVT) (*P* < 0.001) were observed within the observation group, but between-group comparisons showed no statistical significance. No statistically significant differences were observed between groups for gait parameters and spinal mobility measurements (*P* > 0.05). The combined treatment showed favorable safety (8.00% vs. 14.46%, *P* = 0.37) and significantly better quality of life outcomes (*P* < 0.001). Significant Group × Time interactions were found for Cobb angle (*F* = 8.93, *P* < 0.001, η^2^ = 0.214) and CPEI (*F* = 12.56, *P* < 0.001, η^2^ = 0.287), surviving Bonferroni correction.

**Conclusion:**

This study is the first to report the short-term effects of balanced jar therapy combined with the FCAT for AIS. The combined therapy was associated with significant improvement in several scoliosis indices, plantar pressure, and mental health, suggesting potential clinical benefit. However, clinical translation of this treatment modality needs to be optimized and validated in long-term studies.

## Introduction

Scoliosis is a three-dimensional structural deformity of the spine, with adolescent idiopathic scoliosis (AIS) being the most common type, accounting for about 80% of cases (1). AIS occurs most often in the preadolescent period, and the curvature of scoliosis is likely to be aggravated due to the rapid progression of puberty, and the presence of unequal forces on both sides of the spine. Severe scoliosis can affect the development of the thoracic silhouette and height, and may also compress the spinal cord or nerves, potentially leading to paraplegia or spinal stenosis (2, 3). Additionally, physical deformity can cause psychological distress, including low self-esteem and anxiety, which profoundly affects adolescents' well-being (4).

Recent research indicates that AIS is a multifactorial disorder involving genetic susceptibility, neuromuscular imbalance, asymmetric growth modulation, and deficits in postural and sensorimotor control, rather than a purely structural deformity (5). Evidence suggests that AIS is associated with impairments in sensory integration and sensorimotor control, including altered sensory reweighting and proprioception-related deficits, which may contribute to postural instability and asymmetric loading (6–8). This broader pathophysiological understanding supports the clinical rationale for conservative strategies that address not only mechanical alignment but also functional aspects of postural control (8). Current treatment options for AIS include conservative and surgical approaches. Surgery is generally recommended only for severe or rapidly progressive curves with a Cobb angle >45, while most adolescents do not meet surgical indications and therefore receive conservative management (9). Conservative treatment methods currently used in clinical practice include orthopedic braces, physical therapy such as abdominal strengthening, and traditional Chinese medicine (TCM) such as tuina massage and acupuncture (10, 11).

Orthopedic brace therapy is widely recognized as an effective conservative treatment for AIS. Bracing aims to correct spinal deformity by restricting abnormal motion and providing external stabilization (12). A flexible brace is a support device made of elastic material, with flexibility, comfort and adaptability as its main claims, which not only increases the mobility of human joints in a controlled range, but also reduces the restriction of respiration. The Spine Cor Dynamic Flexible Brace has been proved to have a very good therapeutic effect on the patients with Risser's grading of up to grade 3 (13). Jang et al. (14) revealed that thoracolumbar flexible brace has a high rate of correction (36.9%) and has the potential to reduce fixed deformity in patients with AIS. A previous study showed that brace effectiveness is strongly dependent on in-brace correction (IBC) and adherence, with higher initial correction ratios predicting better long-term stabilization (15). Therefore, the flexible brace in the Flexible Corrective Appropriate Technique (FCAT) has greater promise in patients with AIS compared to the rigid brace as it reduces the excessive restriction of joint movement in patients with AIS.

In recent years, increasing evidence has supported the use of multimodal conservative treatments for AIS, particularly the combination of bracing with physiotherapeutic scoliosis-specific exercises (PSSE), which has been shown to improve trunk symmetry, posture, and curve stability more effectively than bracing alone. Prospective studies report that up to 80%−90% of patients receiving combined therapy avoid progression exceeding 5° (16). Additionally, several studies indicate that TCM-based external therapies combined with physical therapy may yield superior outcomes compared with physical therapy alone (17). Balanced jar therapy is a TCM technique that integrates cupping, scraping (gua sha), and manual manipulation, with the goal of improving circulation and muscle tone. Although primarily used for lumbar disc herniation and other chronic musculoskeletal conditions, there is no published research examining its application in AIS. However, Wei et al. (18), found that a 12-month program combining massage and acupoint therapy improved scoliosis progression, suggesting a potential therapeutic value for balanced jar therapy.

Bracing is a cornerstone conservative treatment for AIS, with its primary therapeutic target being mechanical control of curve progression and spinal alignment through external corrective forces (19). Dynamic or flexible brace concepts (e.g., SpineCor) have been studied in AIS and may provide curve control in selected patients, although effectiveness varies across studies and depends on appropriate indications and adherence (20, 21). Cupping-based manual therapies are commonly proposed to influence local microcirculation and soft-tissue mechanical properties, with experimental studies reporting increased local blood flow and reductions in muscle stiffness under specific negative-pressure parameters (22, 23). Although evidence on balanced jar therapy specifically in AIS remains limited, a randomized controlled trial has reported clinical benefits of a combined TCM therapy for AIS, supporting continued investigation of adjunctive TCM-based modalities within conservative care frameworks (18). From a conceptual standpoint, FCAT and balanced jar therapy target different but potentially complementary aspects of AIS: FCAT primarily addresses structural alignment through external corrective forces, whereas balanced jar therapy is hypothesized to influence functional components related to paraspinal muscle tone and sensorimotor control. Based on this distinction, we hypothesized that their combination may lead to differential effects on functional outcomes such as gait and plantar pressure, beyond those achieved by bracing alone. Therefore, this study investigated the efficacy of combining the flexible corrective fitting technique with balanced jar therapy for AIS, with the goal of providing a more comfortable and potentially more effective conservative treatment option.

## Methods

### Study design

This study was a randomized, single-blind study of 106 patients with AIS in a randomized group with the aim of evaluating the improvement of the condition of patients with AIS by the Balance Jar in conjunction with the FCAT. The trial was registered in the Chinese Clinical Trial Registry (ChiCTR2500105010). As an exploratory pilot study, no formal sample size calculation was performed; the sample size was determined based on practical considerations and available resources. Randomization was performed using a computer-generated random number sequence with a 1:1 allocation ratio. Block randomization (block size = 4) was applied to ensure balanced group assignment. Allocation concealment was maintained using sealed, opaque, sequentially numbered envelopes prepared by an independent researcher not involved in enrollment or assessment. The random allocation sequence was generated by this independent researcher, who had no role in participant recruitment, intervention delivery, or outcome assessment. Potential participants were screened and enrolled by attending clinicians at our hospital. After baseline assessments, a research coordinator who was not involved in outcome assessment opened the next sequentially numbered envelope to assign each participant to the control group (FCAT alone) or the observation group (FCAT plus balanced jar therapy). Due to the nature of the interventions, the therapists who delivered the interventions and the participants could not be blinded. However, all outcome assessors and data analysts remained blinded to group allocation throughout the study. As a pilot study investigating a novel combination therapy, no formal sample size calculation was performed. The sample size was determined based on practical considerations and available resources during the study period.

The study was conducted in accordance with the principles of the Declaration of Helsinki, was approved by the hospital ethics committee, and all subjects and their families signed an informed consent form before enrollment.

### Participants

AIS patients who received treatment at our hospital between October 2022 and September 2024 were screened for eligibility and randomly assigned to the control or observation group. Inclusion criteria were: (1) age 10–18 years; (2) radiographically confirmed AIS based on established diagnostic criteria; (3) Cobb angle 10°-40° measured by two independent orthopedic surgeons (ICC = 0.92); (4) skeletal maturity graded as Risser 0–4; (5) leg-length discrepancy < 1 cm. Exclusion criteria: (1) any prior spinal corrective surgery; (2) comorbid spinal conditions such as spondylolisthesis, infection, congenital deformity, tumor, or fracture; (3) systemic diseases affecting bone or neuromuscular function; (4) severe curve progression requiring surgical indication (>45°); (5) coagulation disorders or skin lesions precluding cupping therapy; (6) psychiatric disorders affecting treatment adherence. The CONSORT flow diagram illustrating participant enrollment, allocation, follow-up, and analysis is provided in Supplementary Figure 1.

Each participant received a unique study ID and was randomized according to the pre-generated random sequence. Group assignments were revealed only after baseline evaluation via the concealed envelopes. Data was collected from each group before intervention, 3 months and 6 months after intervention respectively.

### Intervention

**Figure 1:**
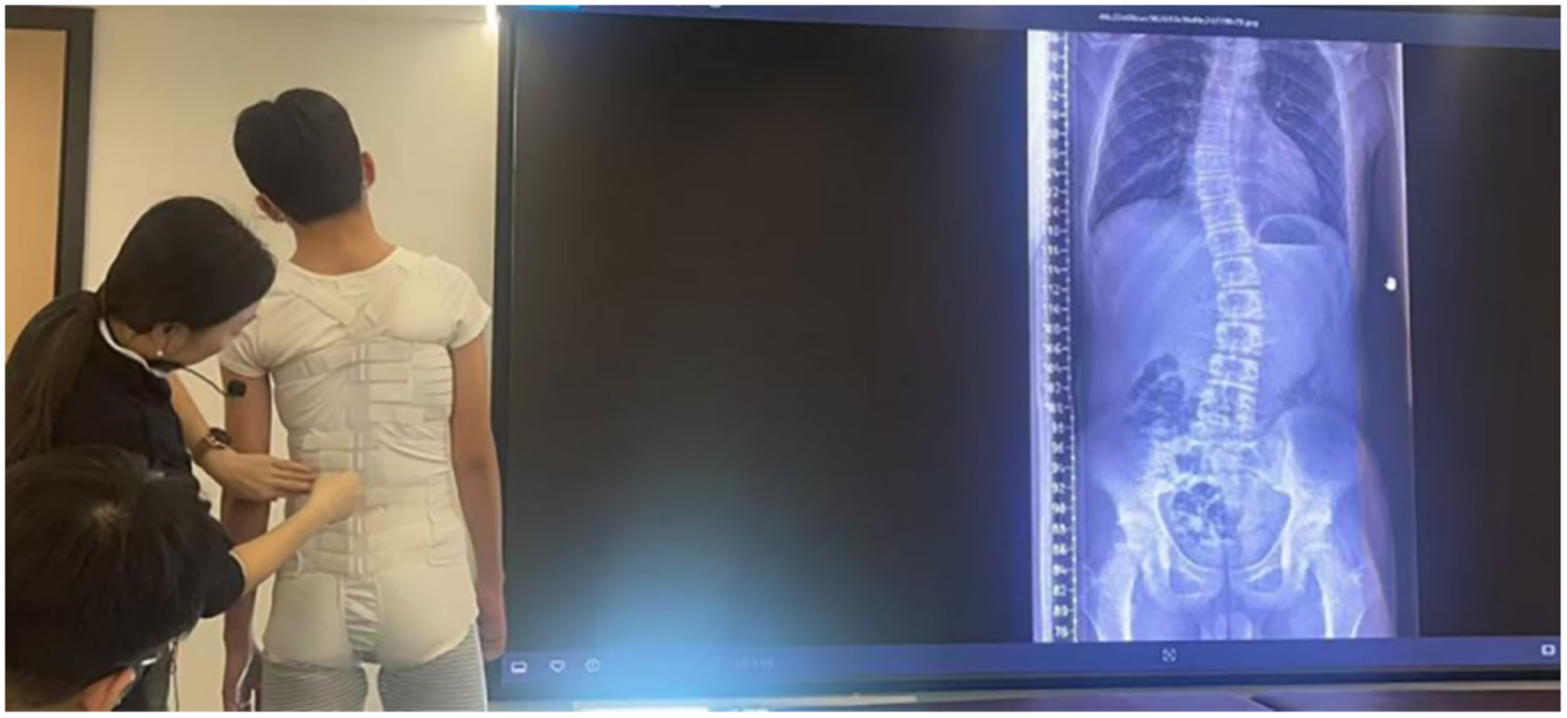
Schematic illustration of the flexible corrective brace used in the flexible corrective appropriate technology (FCAT) intervention.

Both groups received FCAT, which consists of a corrective orthosis supported by a flexible brace. The flexible brace used in this study was designed to modulate whole-body perception, postural control, and muscle movement sensation through mechanical stimulation, thereby improving postural regulation and movement perception (Figure 1). The brace was individually fitted to ensure close contact with the body, and all straps were correctly secured to prevent displacement. During the first 2 weeks, the brace was worn 3–4 times per day for 0.5–1 h per session and removed at night. After 2 weeks, wearing time gradually increased to 4 h per session, and from the third week onward, patients were instructed to wear the brace for approximately 20 h per day, with each removal period not exceeding 2 h. Skin integrity (pain, erythema, swelling, numbness, or other discomfort) was monitored throughout. If significant discomfort or allergic reactions occurred, brace use was suspended and reintroduced after symptom resolution. The planned duration of brace use was at least 6 months and could be extended when clinically indicated.

In addition to FCAT, patients in the observation group received standardized balanced jar therapy based on traditional Chinese medicine. For each session, the patient was placed in the prone position with the back fully exposed. Flash cupping was first applied along both sides of the Bladder meridian on the back for three passes in a clockwise direction using a “stay–pull–stay” technique with light and rapid movements. Once adequate warmth was achieved, kneading cupping was performed along the Du (Governing) Vessel and Bladder meridian for three back-and-forth passes. After kneading, a small amount of lubricating oil was applied, and pushing cupping was conducted along the Du Vessel and Bladder meridian for three passes with moderate negative pressure, moving from the midline toward both sides while avoiding bony prominences and aiming for mild erythema. Finally, shaking cupping was performed for three back-and-forth passes, and residual oil was wiped off. Retention cupping was then applied at Dazhui (GV14) and bilateral Bladder meridian points over the thoracic spine for 5–10 min until the skin appeared red or purple. Throughout the procedure, the therapist monitored cup adhesion and skin color and inquired about patient discomfort; treatment was stopped immediately if severe pain, dizziness, or other adverse symptoms occurred. After cup removal, the skin was cleaned with sterile gauze, and local reactions such as erythema or blistering were documented.

To ensure consistency and reproducibility, the balanced jar therapy was administered under a standardized protocol with the following technical parameters: Cup Type and Material: Medical-grade silicone cups (Hwato, Model: SQZ-1) in diameters of 3, 5, and 6 cm were used. Cup size was selected based on the patient's body size and the specific treatment area. Pressure levels: negative pressure was applied and maintained within a range of −200 to −300 mmHg, monitored by integrated pressure gauges. The pressure was titrated based on individual patient tolerance, with the therapeutic endpoint defined as the appearance of uniform erythema without petechiae or patient report of sharp pain. Duration standardization: Each component of the technique was strictly timed: flash cupping was applied for 2 min per side, kneading cupping for 3 min along each meridian, pushing cupping for 4 min in total, and retention cupping for a fixed duration of 5–10 min at the Dazhui (GV14) and bilateral Bladder Meridian points. The total hands-on treatment time per session was standardized to 25–35 min. Operator variability control: all therapies were delivered by one of three certified TCM practitioners, each with a minimum of 5 years of experience in balanced jar therapy. To minimize inter-operator variability, all therapists participated in a standardized training session and followed a detailed procedural manual prior to the study. Treatment fidelity was assessed through weekly review sessions and random video recordings evaluated by a senior TCM specialist (F.Z.). Adverse Event Management: the protocol mandated immediate cessation of treatment upon patient report of severe pain, dizziness, or the observation of skin damage beyond mild erythema. All such events were documented using a standardized form.

Throughout treatment, particular attention was paid to patients' skin condition and tolerance, and all procedures were performed gently to avoid excessive friction or tissue damage. Participants did not receive additional physiotherapy, structured exercise programs, or other concurrent scoliosis-specific interventions during the study period to isolate the effects of FCAT with or without balanced jar therapy.

### Outcomes

In this study, primary outcomes included the reduction in Cobb angle and apical transverse rotation (ATR), were assessed at 3- and 6-months post-intervention. Secondary outcomes included gait parameters, plantar pressure measures, spinal mobility, adverse events, and quality of life, were also evaluated at the same timepoints.

1. Clinical efficacy. Clinical efficacy at 6 months was evaluated based on Cobb angle and clinical symptoms and categorized into four grades: Cured-Cobb angle < 10°; Markedly effective-Cobb angle reduced by >5° with marked symptom improvement; Effective-Cobb angle reduced by ≤ 5° with symptom improvement; Ineffective-Cobb angle unchanged or increased, with no improvement or worsening of symptoms. The total effective rate was calculated as (cured + markedly effective + effective)/total × 100%.
2. Radiographic indicators of scoliosis. Standard standing full-spine radiographs were obtained at baseline and at 2 weeks, 3 months, and 6 months after the start of treatment, as described previously (24). The following parameters were measured: Cobb angle, ATR, apical vertebral rotation (AVR), and apical vertebral translation (AVT) from the sacral midline.
3. Spinal appearance was assessed using the Spinal Appearance Questionnaire (SAQ) (25) at baseline and at 6 months. The SAQ consists of two parts (pictorial and textual) covering different degrees of deformity, with 20 items across 9 subscales, each scored on a 5-point scale, yielding a total of 100 points. Higher scores indicate poorer satisfaction with appearance.
4. Gait and plantar pressure parameters. Gait and plantar pressure were evaluated using the Footscan plantar pressure testing system under natural walking conditions at baseline and at 2 weeks, 3 months, and 6 months after intervention (26). The primary parameters included gait speed, total support phase as a percentage of the gait cycle, and the center of pressure excursion index (CPEI), measured over at least one complete gait cycle.
5. Spinal mobility. As shown previously (27), patients' spinal mobility was assessed using The Spinal Mouse1 System (Idiag, Fehraltorf, Switzerland) before, 2 weeks, 3 months and 6 months after the intervention. Briefly, measurements were taken from the spinous process of C7 to S3, maximal flexion and maximal extension in the sagittal plane (SP), and maximal right and left lateral flexion positions in the frontal plane (FP), respectively, and the total motion was calculated for SP and FP.
6. Adverse events. Adverse events occurring within 6 months of treatment initiation were recorded, including skin lesions, bleeding, soft-tissue injury, dizziness and fatigue, and joint pain.
7. Quality of life (QOL). Health-related quality of life was assessed using the Scoliosis Research Society-22 (SRS-22) questionnaire before treatment and at 3 and 6 months after treatment. The SRS-22 includes five domains-functional activity, pain, self-image, mental health, and satisfaction with treatment-with 22 items in total, each scored on a 5-point Likert scale. Higher scores reflect better quality of life.
  Radiologic Measurements: all radiographic parameters were assessed by two independent orthopedic surgeons who were blinded to group assignment. Inter-rater reliability was excellent, with intra-class correlation coefficients (ICC) of 0.92 for Cobb angle and 0.88 for ATR measurements.

### Statistical analysis

In order to test whether there was any difference in the variables between the two groups at baseline, independent-samples *t*-tests were used for continuous variables and χ^2^ tests were used for categorical variables. Continuous data are presented as mean ± standard deviation (SD), and categorical data are presented as counts and percentages. For longitudinal outcomes measured at baseline, 2 weeks, 3 months, and 6 months, we used Generalized Least Squares (GLS) models to evaluate Group, Time, and Group × Time interaction effects. GLS was chosen instead of linear mixed-effects models because our primary interest was in estimating marginal (population-average) group-by-time effects rather than subject-specific trajectories, and the relatively small sample size with only four observation timepoints makes additional random-effects specification unlikely to materially change the estimates while increasing model complexity.

For the GLS models, a compound symmetry (CS) covariance structure was specified for the within-subject covariance matrix, assuming constant variance and equal correlations across timepoints. Model residuals were examined and did not reveal major violations of this assumption. The GLS models were fitted using maximum likelihood and included all available repeated measurements; in this trial, complete follow-up data were obtained for all 106 participants at all time points, so no data imputation was required and no special missing-data procedures were applied.

Because nine repeated-measures outcomes were analyzed in the GLS framework, a Bonferroni-adjusted significance level of α = 0.05/9 = 0.0056 was used to control the family-wise error rate. For these outcomes, we therefore distinguish between results that remain statistically significant after Bonferroni correction (adjusted *P* < 0.0056) and nominal trends where the unadjusted *P*-value is < 0.05 but the adjusted *P*-value is ≥ 0.0056. Unless otherwise specified, two-sided *P* < 0.05 was considered statistically significant. All 106 randomized participants were included in the primary analyses according to an intention-to-treat principle, and because there were no losses to follow-up or post-randomization exclusions, the intention-to-treat and per-protocol populations were identical. No formal interim analyses or statistical stopping rules were prespecified for this pilot trial. All statistical analyses were performed using SPSS version 21.0 (IBM Corp., Armonk, NY, USA).

## Results

### Basic characteristics of included patients

A total of 106 adolescents with AIS were enrolled in the study. Four patients did not complete the 6-month follow-up (3 in the control group and 1 in the observation group). The overall mean age was 13.66 ± 1.26 years, and 61.76% of the patients were female. More than half of the participants had a Risser grade of 0, indicating that most patients were still skeletally immature. At baseline, there were no statistically significant differences between the two groups in age, sex distribution, body mass index, Risser stage, curve magnitude (Cobb angle and ATR), or other key clinical variables (all *P* > 0.05), and the groups were therefore considered comparable at baseline (Table 1).

**Table 1:** Baseline characteristics of AIS patients.

**Parameter**	**Control (*n* = 50)**	**Observation (*n* = 52)**	***X*^2^(*t*)**	***P***
Age (years), mean ±SD	13.71 ± 1.05	13.48 ± 1.17	1.043	0.299
**Gender**, ***n*** **(%)**			0.589	0.443
Male	21 (42.00)	18 (34.62)		
Female	29 (58.00)	34 (65.38)		
BMI (kg/m^2^), mean ±SD	19.26 ± 2.51	18.87 ± 2.25	0.827	0.410
**Risser grade**, ***n*** **(%)**			2.056	0.725
0	27 (54.00)	30 (57.69)		
1	6 (12.00)	4 (7.69)		
2	4 (8.00)	3 (5.77)		
3	9 (18.00)	13 (25.00)		
4	4 (8.00)	2 (3.85)		
**Types of scoliosis**, ***n*** **(%)**			0.315	0.854
Thoracic	19 (38.00)	17 (32.69)		
Thoracic and lumbar	15 (30.00)	17 (32.69)		
Lumbar	16 (32.00)	18 (34.62)		
Pre-treatment cobb angle (°), mean ±SD	29.18 ± 4.65	29.76 ± 4.32	0.653	0.515
Pre-treatment ATR angle (°), mean ± SD	12.78 ± 2.25	12.55 ± 2.63	0.505	0.637
Pre-treatment AVT (mm), mean ± SD	25.33 ± 6.37	26.16 ± 7.22	0.615	0.54
Pre-treatment AVR (°), mean ± SD	1.27 ± 0.64	1.34 ± 0.52	0.607	0.545
Age of diagnosis (years), mean ± SD	12.23 ± 1.69	11.76 ± 1.86	1.334	0.185
Weight (kg), mean ± SD	42.74 ± 4.32	41.89 ± 4.88	0.93	0.355
Height (cm), mean ± SD	151.15 ± 7.54	150.59 ± 8.87	0.343	0.732

Values are presented as mean ± SD or *n* (%). Comparisons between groups were conducted using independent-samples *t*-tests for continuous variables and χ^2^ tests for categorical variables. AIS, adolescent idiopathic scoliosis; BMI, body mass index; AVT, apical vertebral translation; AVR, apical vertebral rotation; ATR, angle of trunk rotation; SD, standard deviation.

### Clinical efficacy

**Figure 2:**
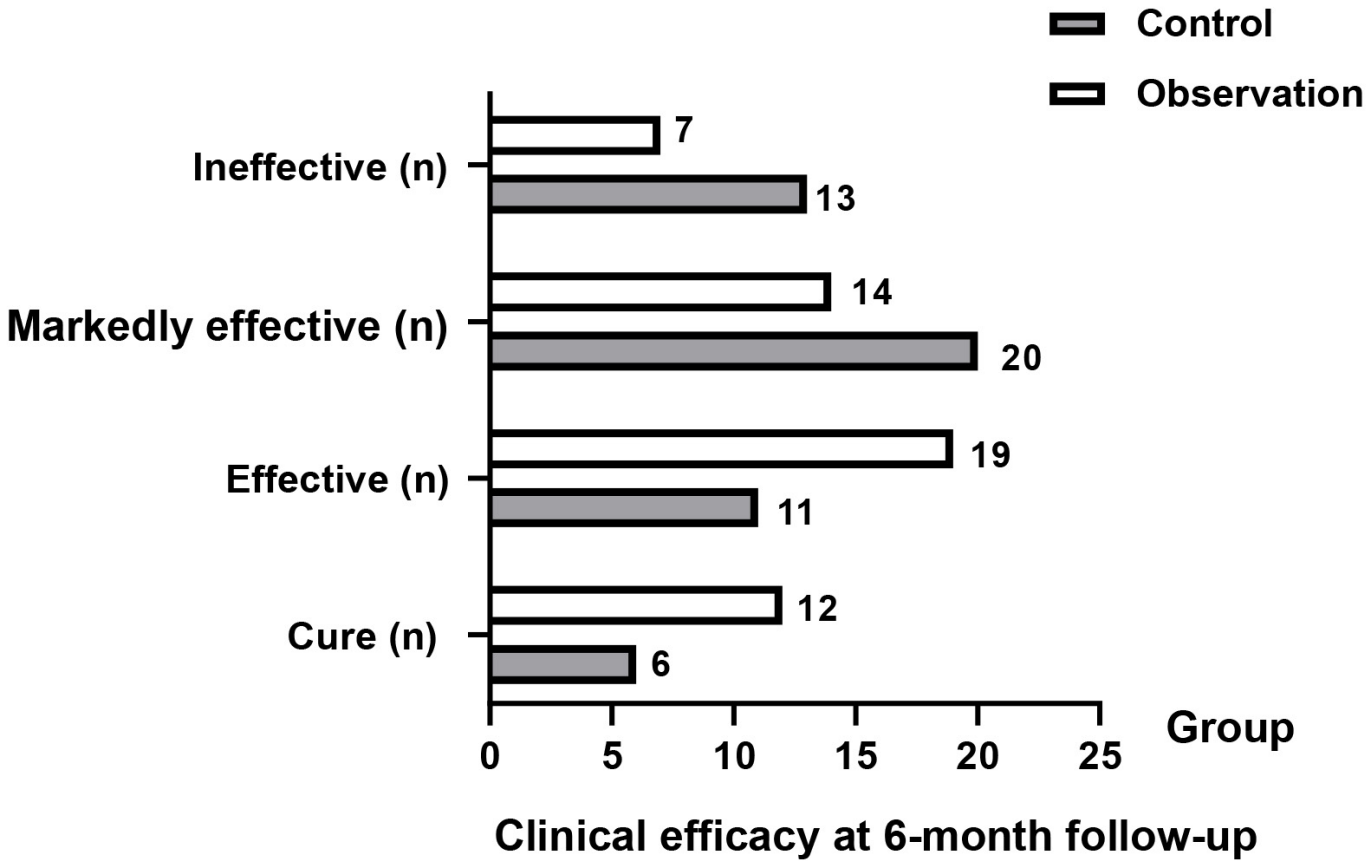
Clinical efficacy outcomes at 6 months in the control and observation groups. Categories include cured, markedly effective, effective, and ineffective. Total effective rate = (cured + markedly effective + effective)/total × 100%.

In terms of clinical efficacy, 74% of the patients in the control group were assessed as effective, and 86.54% of the patients in the observation group were effectively treated. Although the efficacy of the two groups was not statistically significant, the effective rate of the observation group showed a non-significant trend of increasing compared with the control group (*P* = 0.111, Figure 2).

### Spinal deformity parameters

**Figure 3:**
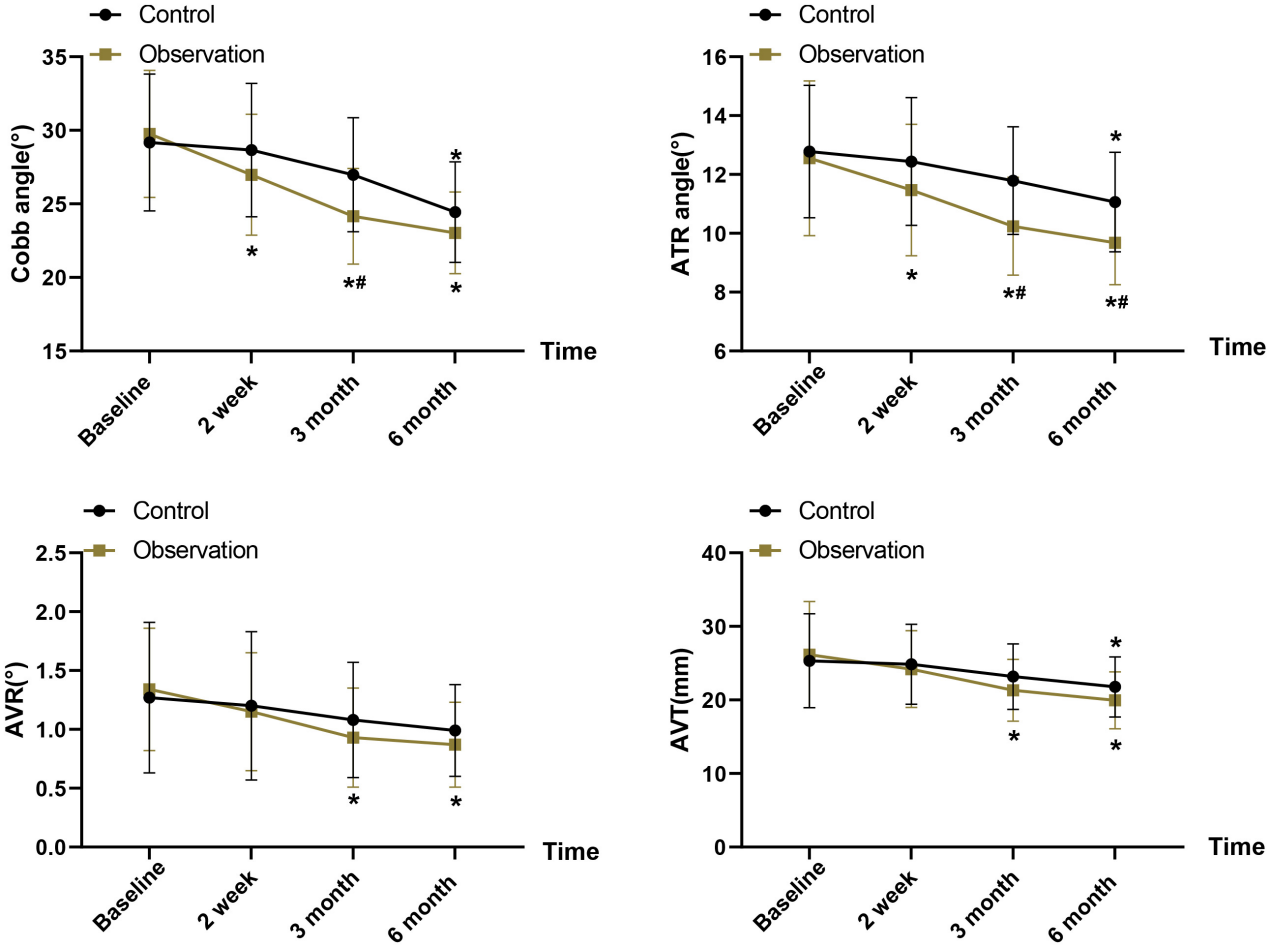
Changes in radiologic parameters [Cobb angle, angle of trunk rotation (ATR), and center of pressure excursion index (CPEI)] at baseline (T0), 3 months (T1), and 6 months (T2). The data are presented as mean ± standard deviation (SD). *P < 0.05 vs. baseline within the same group; *^#^*P* < 0.05 vs. control group at the same timepoint.

Further analyzing the changes of scoliosis indexes in the two groups after the intervention, the Cobb angle and ATR of both groups showed reduction after 6 months of treatment, and compared with the control group, the Cobb angle (24.16 ± 3.25 vs. 26.98 ± 3.87, *P* = 0.007) and ATR (10.24 ± 1.66 vs. 11.79 ± 1.83, *P* = 0.003) were significantly reduced after 3 months of intervention in the observation group. For AVR and AVT, between-group comparisons did not reach statistical significance, though the observation group showed changes in AVR (*P* = 0.001) and AVT (*P* < 0.001) compared to their own baseline measurements (Figure 3). The GLS analysis revealed a statistically significant Group × Time interaction for Cobb angle after Bonferroni correction (mean difference: −2.82°, 95% CI: −4.35 to −1.18; *F*(3,300) = 8.93, *P* < 0.001, partial η^2^ = 0.214). The observation group demonstrated substantially greater reduction in spinal curvature across all timepoints compared to control. Similarly, ATR showed a significant interaction effect, though this did not survive strict Bonferroni correction [*F*(3,300) = 6.45, *P* = 0.002, partial η^2^ = 0.187] (Supplementary Table 1).

From a clinical standpoint, the mean Cobb angle decreased by 4.74° in the control group (from 29.18° to 24.44°) and by 6.73° in the observation group (from 29.76° to 23.03°) over 6 months, corresponding to relative reductions of approximately 16% and 23%, respectively; given that the standard measurement error for Cobb angle is typically 3°-5° and that a ≥5° change is widely regarded as indicating true curve progression or improvement, these reductions likely represent clinically meaningful structural benefits, with the combined therapy providing an additional ≈2° of correction beyond bracing alone. Likewise, the ATR reduction was 1.72° in the control group vs. 2.87° in the observation group, which is close to or slightly above reported scoliometer measurement precision and consistent with a visibly reduced trunk rotation in the combined-treatment group.

### SAQ score

**Figure 4:**
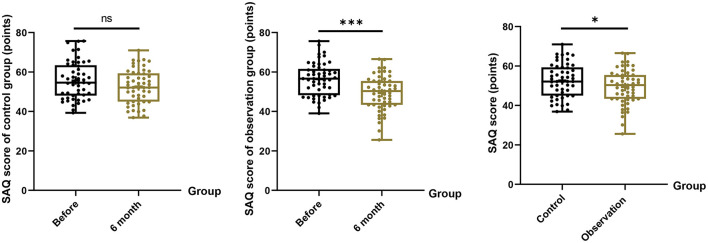
Spinal appearance questionnaire (SAQ) scores before and 6 months after intervention for both groups. Lower scores indicate improved appearance perception. *P < 0.05; ***P < 0.001 vs. baseline.

Compared with the control group, the SAQ scores of patients in the observation group were noticeably lower after 6 months of correction, indicating that the patients and their families were more satisfied with the effect of spinal correction (49.32 ± 8.86 vs. 52.71 ± 8.47, *P* = 0.049, Figure 4).

### Gait and plantar pressure measurements

**Figure 5:**
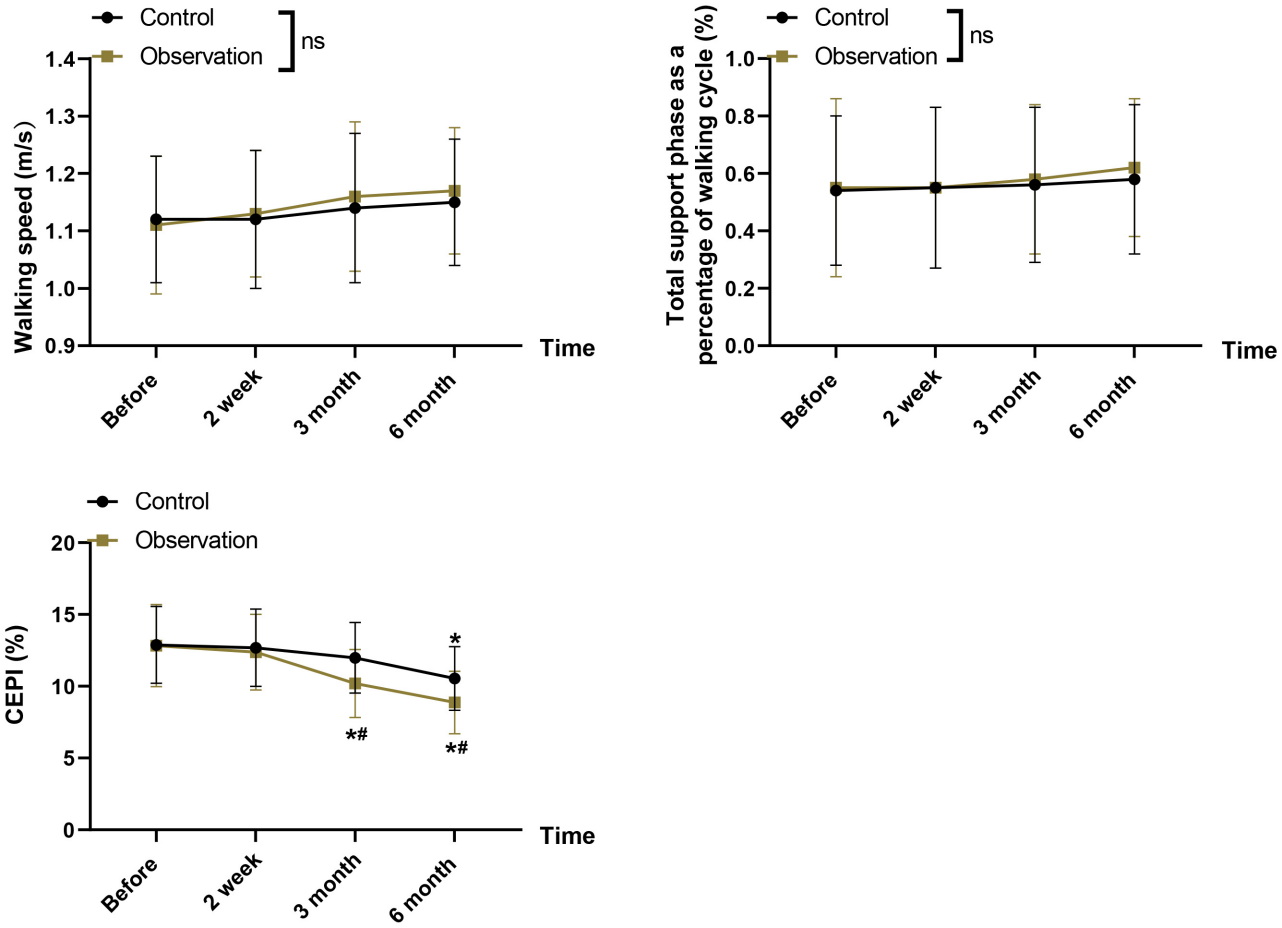
Gait and plantar pressure parameters-including walking speed, total support phase, and center of pressure excursion index (CPEI)-before and after intervention. Error bars represent mean ± SD. *^#^*P* < 0.05 vs. control group at the same timepoint.

There was no statistically significant difference between the two groups in terms of step speed and total support phase as a percentage of walking cycle (*P* > 0.05), indicating that gross spatiotemporal gait parameters were largely preserved in both groups. There was a significant decrease in CPEI after 6 months of correction in both groups, and the decrease in CPEI was greater in the observation group compared to the control group (8.87 ± 2.17 vs. 10.54 ± 2.21, *P* = 0.020, Figure 5). CPEI demonstrated a moderate-to-large Group × Time effect (partial η^2^ = 0.287), with a between-group difference at 6 months of −1.67 (95% CI: −2.87 to −0.48) (Supplementary Table 1). Although no formal MCID has been established for CPEI or other plantar-pressure indices in AIS, prior studies show that adolescents with scoliosis typically exhibit only small absolute but consistent asymmetries in plantar-pressure distribution relative to healthy controls; thus, a nearly 4-point reduction in CPEI in the observation group (vs. 2.34 in the control group) suggests a shift toward more symmetric loading and improved postural control, even if the effect is modest in absolute terms. The observation group showed marked functional improvement over the 6-month intervention period, while the control group exhibited minimal change.

### Spinal mobility assessment

**Figure 6:**
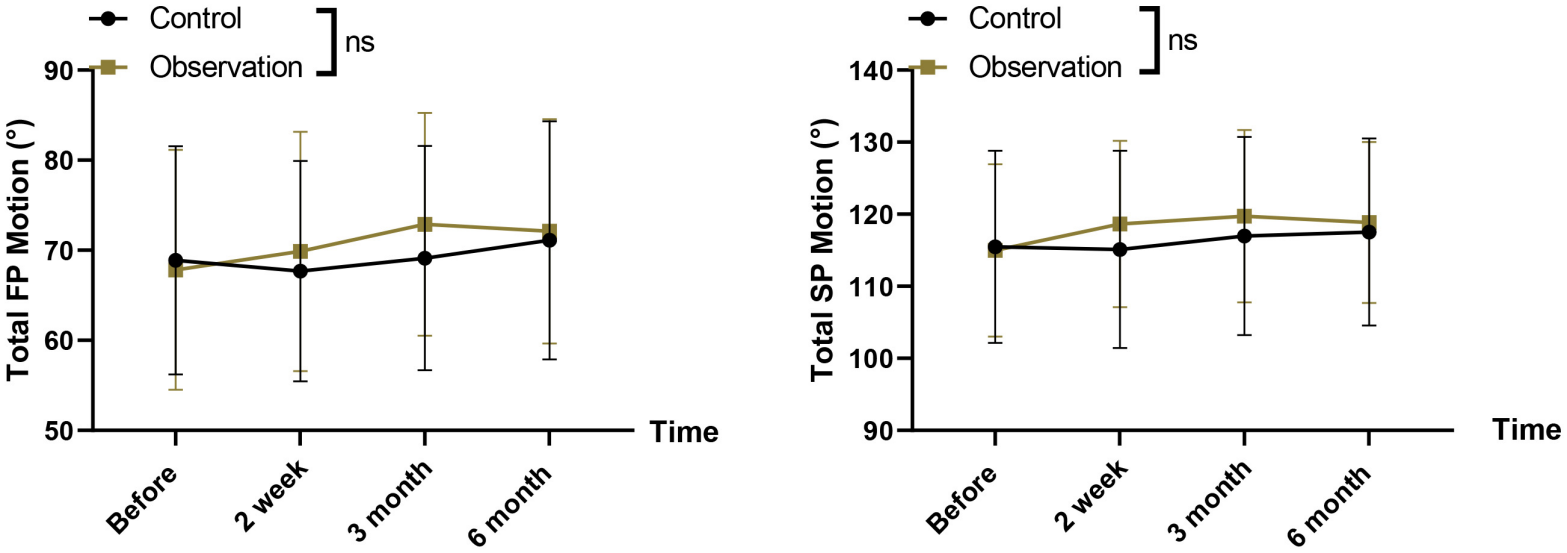
Spinal mobility assessments-including total frontal-plane (FP) motion and total sagittal-plane (SP) motion-before and after intervention.

In terms of spinal mobility assessment, there was no statistically significant difference in total FP (control, *P* = 0.988; Observation, *P* = 0.680) and total SP (control, *P* = 0.992; Observation, *P* = 0.761) exercise at 6 months post-intervention compared to pre-intervention in both groups (Figure 6), and in the balanced canisters At 3 months after administration of the treatment, there was a trend toward an increase in total FP and total SP movements, suggesting that the balance canister may increase spinal mobility in AIS, whereas in the short term, no improvement in spinal mobility could be observed with the flexible corrective fit technique. Although several gait parameters showed time-related improvements, none demonstrated significant Group × Time interactions after Bonferroni correction. FP total motion [*F*(3,300) = 3.45, *P* = 0.017] and SP total motion [F(3,300) = 2.89, *P* = 0.036] showed nominal significance but did not meet the corrected threshold (Supplementary Table 1).

### Adverse effects

There was no significant difference in the incidence of adverse events between the two groups (8.00% vs. 14.46%, *P* = 0.37), indicating that the combination of balanced jar therapy with flexible corrective appropriate technology was well tolerated and demonstrated a high level of safety (Table 2). The recorded adverse events were limited to mild skin lesions (erythema or small ecchymoses), transient dizziness or fatigue, and transient joint discomfort. No infections, neurovascular complications, or other serious adverse events occurred in either group, and no patient discontinued brace treatment or balanced jar therapy because of adverse events. In most cases, symptoms resolved spontaneously or within a few days after brief treatment interruption or simple local care. Quality of life, assessed using the SRS-22 questionnaire, improved markedly in both groups after the intervention, with significantly greater improvement observed in the observation group compared with the control group (*P* < 0.001, Table 3).

**Table 2:** Adverse events statistics for AIS patients in both groups over a 6-month period, *n* (%).

**Groups**	**Skin lesions**	**Bleeding**	**Soft tissue injury**	**Dizziness and fatigue**	**Joint pain**	**Total rates**
Control (*n* = 50)	2 (4.00)	0 (0.00)	0 (0.00)	0 (0.00)	2 (4.00)	4 (8.00)
Observation (*n* = 52)	2 (3.85)	1 (1.92)	1 (1.92)	2 (3.85)	1 (1.92)	7 (13.46)
*X* ^2^						0.79
*P*						0.37

SD, standard deviation; *n*, number of participants.

**Table 3:** Comparison of SRS-22 scores 6 months after correction between the two groups (mean ± SD).

**Groups**	**Functional activity**		**Self-image**		**Mental health**		**Pain**		**Treatment satisfaction**		**Totals**	
	**Before**	**6 months**	**Before**	**6 months**	**Before**	**6 months**	**Before**	**6 months**	**Before**	**6 months**	**Before**	**6 months**
Control (*n* = 50)	4.21 ± 0.52	4.52 ± 0.48a	3.88 ± 0.62	4.07 ± 0.54	3.72 ± 0.55	4.11 ± 0.54a	4.18 ± 0.46	4.43 ± 0.44a	3.62 ± 0.58	4.52 ± 0.61a	19.23 ± 1.17	20.62 ± 0.93a
Observation (*n* = 52)	4.17 ± 0.43	4.89 ± 0.51a	3.81 ± 0.56	4.33 ± 0.52a	3.82 ± 0.59	4.49 ± 0.43a	4.14 ± 0.48	4.69 ± 0.41a	3.65 ± 0.53	4.87 ± 0.56a	19.31 ± 1.05	22.12 ± 0.89a
*t*	0.424	3.77	0.599	2.477	0.885	3.939	0.429	3.089	0.273	3.020	0.364	8.324
*P*	0.672	< 0.001	0.551	0.015	0.379	< 0.001	0.669	0.002	0.786	0.003	0.717	< 0.001

Values are presented as mean ± SD. Within-group differences were examined using paired-samples *t*-tests, and between-group differences were tested using independent-samples *t*-tests. ^a^*P* < 0.05 compared with baseline within the same group.

Higher SRS-22 scores indicate better health-related quality of life. SRS-22, scoliosis research society-22 questionnaire; SD, standard deviation; *n*, number of participants.

## Discussion

Our randomized, single-blind pilot study suggests that the addition of balanced jar therapy to a flexible corrective brace was associated with greater short-term improvements in scoliosis indices (notably Cobb angle and ATR) and plantar pressure distribution compared with flexible bracing alone. The magnitude of the observed changes may be clinically relevant, with both groups achieving Cobb angle reductions that exceed typical radiographic measurement error, and the combined therapy providing an additional ≈2° of correction and a ≈23% relative reduction from baseline. ATR and CPEI showed consistent changes with reduced trunk rotation and more symmetric plantar loading, though formal MCIDs for these measures have not been established. These results are consistent with the growing body of recent evidence supporting multimodal conservative strategies for AIS, which emphasize combining mechanical correction with targeted neuromuscular or sensorimotor interventions to optimize early curve response and patient-centered outcomes.

Brace efficacy depends critically on wear-time and adherence; recent work shows that early adherence strongly predicts subsequent brace wear and long-term treatment success, making early monitoring and adherence support a priority in clinical practice (28). Furthermore, flexible or soft braces can improve comfort and mobility but may provide less corrective force than rigid designs; recent comparative evaluations indicate that soft-brace concepts can still achieve meaningful control of curve progression in selected patients, particularly when used within individualized, multimodal treatment programs (29). Our results suggest that early curve improvement when balanced jar therapy was added, and are consistent with the idea that adjunctive interventions may augment the corrective environment provided by a flexible brace (10).

AIS is associated with altered sensorimotor control, asymmetric loading and measurable changes in gait and plantar-pressure metrics. Plantar-pressure and center-of-pressure metrics have been used to characterize functional asymmetry in AIS (30, 31). From a mechanistic standpoint, functional measures such as plantar-pressure indices may respond to changes in postural control and loading symmetry over shorter time frames, even when radiographic structural changes are modest. From a mechanistic standpoint, functional measures such as plantar-pressure indices may respond to changes in postural control and loading symmetry over shorter time frames, even when radiographic structural changes are modest (6, 30). The improvements in CPEI and other plantar-pressure parameters observed in the combined-treatment group suggest a plausible underlying mechanism: balanced jar therapy might reduce paraspinal muscular asymmetry and improve sensorimotor feedback, which in turn could support a more symmetric stance and gait when used alongside a corrective brace (32).

High-quality randomized controlled trial (RCTs) and systematic reviews have recently reinforced the benefit of PSS and other sensorimotor programs for improving trunk shape, rotation, and sometimes Cobb magnitude in mild-to-moderate AIS (27). When brace therapy is combined with targeted exercise, outcomes for balance, spinal mobility, and plantar pressure appear to improve relative to bracing alone in several reports, supporting a multimodal care model similar in spirit to our combined intervention (32).

Brace-associated stress and quality-of-life impacts are well documented; recent studies emphasize that early physical improvements and better comfort/adherence strategies can mitigate negative psychosocial effects and improve SRS-22 domains, especially self-image and mental health (33). In our study, the combined therapy was associated with superior improvements in SRS-22 mental health and total scores without increased serious adverse events, which might indicate that the intervention could be both efficacious and acceptable in the short term. However, the study's pilot design, single-blind format, and relatively short follow-up (6 months) limit our ability to infer long-term structural benefits; larger, adequately powered, and longer-duration trials are required (28).

Despite promising results from various conservative modalities, systematic reviews indicate that evidence quality for many adjunctive therapies—including PSSE—is still limited due to heterogeneity in intervention protocols, variability in brace types, and short follow-up durations. More rigorous RCTs with standardized 3D outcomes, functional measures, and patient-reported metrics are needed to clarify independent and synergistic effects across treatment components (34).

Based on current evidence and our findings, future research should (1) integrate objective adherence monitoring (smart-brace sensors and mHealth platforms) to maximize dosage fidelity; (2) combine standardized PSSE or sensorimotor retraining with bracing and adjunctive manual modalities when ethically and practically feasible; (3) include functional endpoints such as plantar-pressure metrics and patient-reported outcomes alongside radiographic measures; and (4) use non-radiative imaging (e.g., validated ultrasound protocols) when appropriate to reduce cumulative radiation exposure during frequent follow-up. These research directions are supported by recent technological and clinical advances and will strengthen the evidence base for multimodal conservative management of AIS (35).

However, there are still limitations. First, this study is a single-blind study, and the sample size is limited, which can lead to reduced statistical power, especially for secondary outcomes. In addition, because we applied a Bonferroni correction across nine repeated-measures endpoints, several secondary outcomes (such as AVT, frontal-plane total motion, and sagittal-plane total motion) showed unadjusted *P*-values slightly below the conventional 0.05 threshold but did not remain statistically significant after adjustment; given the conservative nature of Bonferroni correction and the exploratory status of these endpoints, such borderline findings should be interpreted as hypothesis-generating rather than confirmatory. The conclusions drawn, particularly regarding secondary outcomes, should be interpreted with caution. Second, the 6-month follow-up period is relatively short for a chronic condition like AIS, and the long-term effects of the intervention remain unknown. Third, as a pilot study, no formal sample size calculation was performed, which further affects the statistical power of our findings. The limited sample size and statistical power may account for the non-significant group × time interactions observed in some secondary outcomes. Additionally, the absence of detailed baseline characterization, such as Lenke curve type, apex level, comprehensive maturity indicators (Sanders stage, menarche status), and curve flexibility, represents another significant limitation. These factors may influence treatment response in AIS, and their absence limits the granularity of our subgroup analysis and the generalizability of our findings. Based on the findings of this pilot study, future research should aim to include larger sample sizes with formal power calculations, longer follow-up periods to assess the sustainability of treatment effects, and more comprehensive baseline characterization to identify potential predictors of treatment response. In addition, the study was conducted at a single tertiary TCM hospital in southern China, and all participants were Chinese adolescents with mild-to-moderate AIS treated under a specific flexible brace protocol (FCAT) and a standardized balanced jar therapy program delivered by a small team of highly experienced TCM practitioners. While this controlled context and high level of therapist expertise enhance internal validity and treatment fidelity, they also limit external validity: the observed effects may not fully generalize to AIS populations with different ethnic backgrounds, curve patterns, or severity distributions, to healthcare systems with different resources, reimbursement structures, or cultural attitudes toward TCM and bracing, or to settings where balanced jar therapy is delivered by less experienced clinicians or integrated into different multimodal rehabilitation protocols. Therefore, our findings should be interpreted as preliminary and context-specific, and replication in multi-center trials involving broader patient populations and diverse practice environments will be essential to establish the generalizability and scalability of this combined intervention.

## Conclusion

Our results suggest that balancing jars therapy with flexible corrective fit techniques may be effective in improving scoliosis and plantar pressure in AIS in the short term with fewer adverse effects and a high degree of safety. However, these findings require confirmation in long-term, large-scale studies before clinical translation can be considered. Future work should optimize the intervention protocol and include larger sample sizes.

## Data Availability

The original contributions presented in the study are included in the article/Supplementary material, further inquiries can be directed to the corresponding authors.
