# Supplementary figures and images for: Clinical study on the treatment of adolescent idiopathic scoliosis by balanced jar therapy with flexible corrective appropriate technology
Source: Front Med (Lausanne). 2026 Mar 16;13:1716190. doi: 10.3389/fmed.2026.1716190 (PMC13034793; doi:10.3389/fmed.2026.1716190)

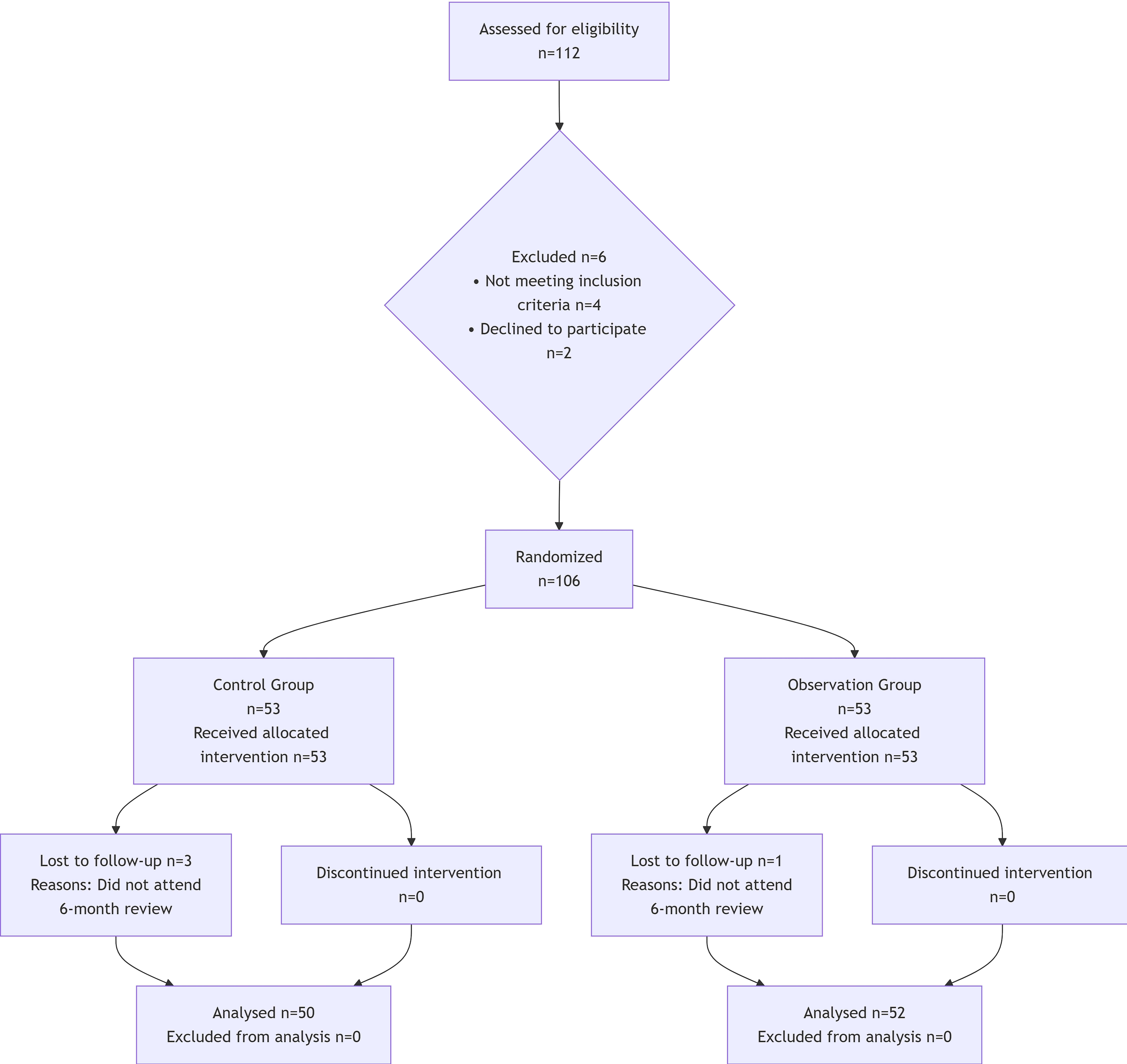

Supplement: Supplementary file 1 [file Data_Sheet_1.pdf]
