# Supplementary material for: Clinical study on the treatment of adolescent idiopathic scoliosis by balanced jar therapy with flexible corrective appropriate technology
Source: Front Med (Lausanne). 2026 Mar 16;13:1716190. doi: 10.3389/fmed.2026.1716190 (PMC13034793; doi:10.3389/fmed.2026.1716190)
Supplement: Supplementary file 2 [file Table_1.docx]

**Supplementary Table 1 Generalized least squares analysis of outcomes**

| **Outcome Measure** |  | **Group Effect F(p)** | **Time Effect F(p)** | **Group × Time F(p)** | **Partial η²** | **Bonferroni Significance** |
| --- | --- | --- | --- | --- | --- | --- |
| **Cobb Angle** |  | F=4.32, p=0.039 | F=58.71, p<0.001 | F=8.93, p<0.001 | 0.214 | **p<0.001*** |
| **ATR** |  | F=6.18, p=0.014 | F=42.56, p<0.001 | F=6.45, p=0.002 | 0.187 | p=0.002 |
| **AVR** |  | F=0.87, p=0.352 | F=15.23, p<0.001 | F=1.24, p=0.295 | 0.089 | p=0.295 |
| **AVT** |  | F=2.15, p=0.144 | F=28.94, p<0.001 | F=3.78, p=0.011 | 0.152 | p=0.011 |
| **Walking Speed** |  | F=1.56, p=0.213 | F=8.67, p<0.001 | F=2.34, p=0.073 | 0.098 | p=0.073 |
| **Total Support Phase** |  | F=0.92, p=0.339 | F=6.45, p=0.002 | F=1.87, p=0.134 | 0.076 | p=0.134 |
| **CPEI** |  | F=9.24, p=0.003 | F=67.83, p<0.001 | F=12.56, p<0.001 | 0.287 | **p<0.001*** |
| **FP Total Motion** |  | F=1.23, p=0.269 | F=4.32, p=0.005 | F=3.45, p=0.017 | 0.121 | p=0.017 |
| **SP Total Motion** |  | F=0.78, p=0.378 | F=5.67, p=0.001 | F=2.89, p=0.036 | 0.103 | p=0.036 |

Notes: Values represent F-statistics and corresponding P-values derived from generalized least squares (GLS) models evaluating Group effects, Time effects, and Group × Time interactions. Partial η² indicates effect size for each interaction term. Bonferroni-adjusted significance was based on α = 0.0056 to account for multiple comparisons.***Significant after Bonferroni correction. Cobb Angle: Magnitude of spinal curvature in degrees; ATR: Angle of trunk rotation; AVR: Apical vertebral rotation; AVT: Apical vertebral translation; CPEI: Center of pressure excursion index; FP Total Motion: Total frontal-plane motion; SP Total Motion: Total sagittal-plane motion; GLS: Generalized least squares; η²: Eta-squared (effect size).
